# Metabolic Profiling Reveals Aggravated Non-Alcoholic Steatohepatitis in High-Fat High-Cholesterol Diet-Fed Apolipoprotein E-Deficient Mice Lacking Ron Receptor Signaling

**DOI:** 10.3390/metabo10080326

**Published:** 2020-08-11

**Authors:** Joselyn N. Allen, Adwitia Dey, Jingwei Cai, Jingtao Zhang, Yuan Tian, Mary Kennett, Yanling Ma, T. Jake Liang, Andrew D. Patterson, Pamela A. Hankey-Giblin

**Affiliations:** 1 Department of Veterinary and Biomedical Sciences, The Pennsylvania State University, University Park, PA 16802, USA; joselyn.allen@nih.gov (J.N.A.); deyadwitia@gmail.com (A.D.); caij@gene.com (J.C.); jingtao.zhang@ntu.edu.sg (J.Z.); yzt11@psu.edu (Y.T.); mjk26@psu.edu (M.K.); 2 Liver Diseases Branch, National Institute of Diabetes and Digestive and Kidney Diseases, The National Institutes of Health, Bethesda, MD 20814, USA; yanling@ma.nih.gov (Y.M.); jakel@bdg10.niddk.nih.gov (T.J.L.)

**Keywords:** Ron receptor tyrosine kinase, macrophage stimulating protein (MSP), non-alcoholic steatohepatitis, lipid metabolism, mass spectrometry, nuclear magnetic resonance

## Abstract

Non-alcoholic steatohepatitis (NASH) represents the progressive sub-disease of non-alcoholic fatty liver disease that causes chronic liver injury initiated and sustained by steatosis and necroinflammation. The Ron receptor is a tyrosine kinase of the Met proto-oncogene family that potentially has a beneficial role in adipose and liver-specific inflammatory responses, as well as glucose and lipid metabolism. Since its discovery two decades ago, the Ron receptor has been extensively investigated for its differential roles on inflammation and cancer. Previously, we showed that Ron expression on tissue-resident macrophages limits inflammatory macrophage activation and promotes a repair phenotype, which can retard the progression of NASH in a diet-induced mouse model. However, the metabolic consequences of Ron activation have not previously been investigated. Here, we explored the effects of Ron receptor activation on major metabolic pathways that underlie the development and progression of NASH. Mice lacking apolipoprotein E (ApoE KO) and double knockout (DKO) mice that lack ApoE and Ron were maintained on a high-fat high-cholesterol diet for 18 weeks. We observed that, in DKO mice, the loss of ligand-dependent Ron signaling aggravated key pathological features in steatohepatitis, including steatosis, inflammation, oxidation stress, and hepatocyte damage. Transcriptional programs positively regulating fatty acid (FA) synthesis and uptake were upregulated in the absence of Ron receptor signaling, whereas lipid disposal pathways were downregulated. Consistent with the deregulation of lipid metabolism pathways, the DKO animals exhibited increased accumulation of FAs in the liver and decreased level of bile acids. Altogether, ligand-dependent Ron receptor activation provides protection from the deregulation of major metabolic pathways that initiate and aggravate non-alcoholic steatohepatitis.

## 1. Introduction

Receptor tyrosine kinases and their downstream targets have long been explored as anti-cancer therapies. Specifically, the Ron tyrosine receptor has been extensively explored as the forefront of anti-cancer therapeutics. In recent years, targeting the Ron receptor has been recognized as a potential treatment strategy for addressing diet-induced metabolic disorders, including non-alcoholic steatohepatitis. This liver specific disease is orchestrated by hepatic and extra-hepatic tissue signaling [1,2]. Primary insults that are released from expanded white adipose tissue triggers ectopic fat accumulation through the deregulation of normal hepatic metabolic function. Exaggerated *de novo* lipogenesis (DNL) and fatty acid uptake concurrent with exhausted lipid handling and elimination pathways play a crucial role in steatohepatitis onset and progression [3,4]. Chronic inflammation is a major hallmark of steatohepatitis that occurs in parallel to steatosis and facilitates the development of secondary insults, such as oxidative stress, endoplasmic reticulum (ER) stress, and other pathological events [5,6,7], which substantially increase the risk of developing cirrhosis, hepatocellular carcinoma (HCC), and mortality. As the second leading etiology for liver failure and liver transplant, NASH, the subtype of the most common chronic liver disorder NAFLD, has become a global epidemic [8]. The unmet demand for FDA approved drugs for treating NASH warrants further exploration of novel anti-NASH therapeutics.

The high affinity surface receptor Recepteur d’Origine Nantais (Ron) modulates macrophage heterogeneity in many highly metabolic tissues [9]. The engagement of the Ron receptor by its ligand, macrophage stimulating protein (MSP), suppresses a pro-inflammatory response in macrophages while favoring the reparative functions of alternatively activated macrophages [9,10,11,12,13,14,15]. It does so by inhibiting nuclear factor NF-κB activation at the level of IκB kinase (IKK) activity and promotes the expression of anti-inflammatory genes through STAT3 (signal transducer and the activator of transcription 3) tyrosine phosphorylation and SOC-1 and SOC-3 (suppressor of cytokine signaling -1 and -3) upregulation [11,13,15]. Several reports have demonstrated the crucial role of tissue-resident macrophages in initiating and exaggerating metabolic diseases using macrophage depletion techniques [16,17,18,19,20]. Accumulating evidence points to the effects of the predominating macrophage phenotype in maintaining tissue homeostasis and curbing disease onset and progression. A significant imbalance of inflammatory (M1) and reparative (M2) macrophages in metabolic tissues, in part, dictates the progressiveness of the metabolic syndrome including adipose tissue dysfunction, insulin resistance, atherosclerosis and non-alcoholic steatohepatitis [21,22,23,24].

Apolipoprotein E knock-out (ApoE KO) mice are a metabolic syndrome model used in cardiovascular and non-alcoholic steatohepatitis research [25]. In humans, the systemic release of oxidative stress and inflammatory mediators in non-alcoholic steatohepatitis significantly contributes to the onset of atherosclerosis [26,27,28,29]. In a previous study [9], we investigated the effects of Ron receptor signaling on macrophage polarization in the pathogenesis of atherosclerosis, as well as non-alcoholic steatohepatitis (NASH) using a high-fat high-cholesterol fed ApoE KO mouse model. Transcriptional profiling of FACS-isolated Ron positive (+) and Ron negative (−) CD45^+^ F4/80^+^ macrophage populations showed distinct phenotypes. The lack of Ron expression (Ron^−^) on both aorta- and liver-resident macrophages had higher expression of pro-inflammatory marker iNOS, which is a well-known indicator of a classically activated (M1) activation. Ron expressing populations (Ron^+^) demonstrated higher expression of arginase-1 (Arg1) which is a well-reported marker for alternatively activated (M2) macrophages. The genetic profiling of FACS-isolated CD11c-negative (−) M2-like and CD11c-positive (+) M1-like macrophage populations showed distinct Ron expression levels. CD11c-negative M1-like macrophages exhibited higher expression of Ron, which further corroborated the favorable effects of Ron signaling on M2 macrophage polarization. In past in vitro studies [11,30,31], we have extensively shown that the activation of Ron by its ligand MSP induces an anti-inflammatory (M2) phenotype while inhibiting the expression of inflammatory mediators including *Tnfα*, *Inos*, and *Il-12b*.

To challenge the function of the Ron receptor using the ApoE KO mouse model, ApoE KO mice deficient for MSP-dependent Ron signaling (double knock-out or DKO) were fed a high-fat high-cholesterol diet. DKO mice demonstrated accelerated atherosclerosis and NASH pathogenesis. Altogether, our past findings suggest that the lack of Ron on tissue-resident macrophages can promote an inflammatory phenotype that, in part, accelerates NASH pathogenesis and may predispose to atherosclerosis, a predisposition commonly seen with human NASH. However, the exacerbated macrophage-mediated inflammation in DKO mice partially explains the accelerated NASH pathogenesis. Because the metabolic changes that underly the accelerated phenotype of DKO mice were not explored, here we revisit this ApoE KO NASH model to investigate the metabolic consequences that arise in response to impaired Ron signaling.

We show that the Ron receptor functions to maintain adipocyte homeostasis, as well as normal liver metabolism that is preventative to NASH development. This study provides further evidence that the Ron receptor may serve as an ideal therapeutic candidate for the treatment of non-alcoholic steatohepatitis and other obesity-associated diseases.

## 2. Results

### 2.1. Loss of Ron Aggravates White Adipose Tissue Metabolic Dysfunction

**Figure 1:**
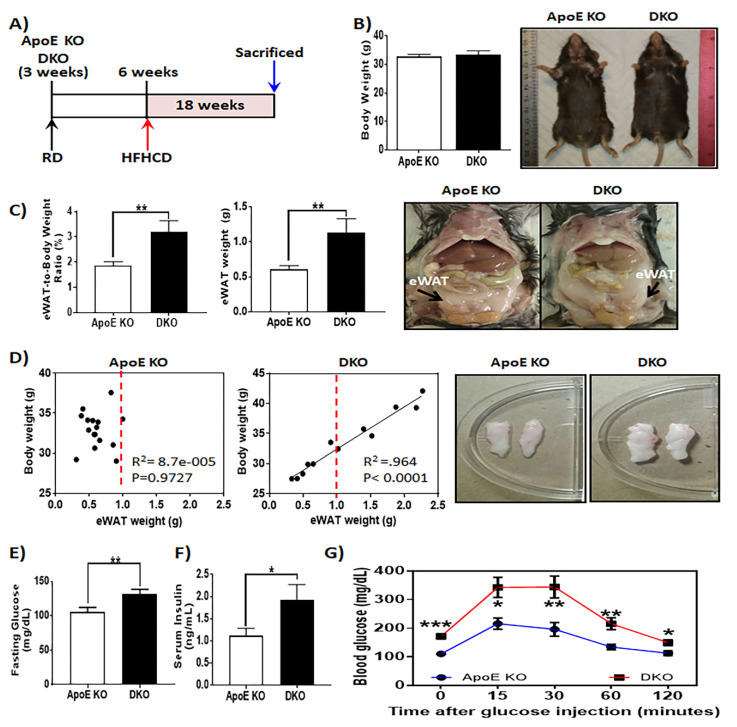
Metabolic phenotype of high-fat high-cholesterol diet (HFHCD)-fed ApoE knockout (KO) and double knockout (DKO) mice. (**A**) Scheme of experimental design shown. Six-week-old mice were maintained on a HFHCD for 18 weeks. (**B**) Effects of impaired Ron receptor signaling on total body weight (representative image shown) and (**C**) epidydimal white adipose tissue mass (representative image shown), *n* = 12–16 per group. (**D**) Correlation plots between measured body weight and epididymal white adipose tissue weights (eWAT) for HFHCD-fed animals (representative image shown), *n* = 12–16 per group. (**E**) Blood glucose concentration measured in mice fasted for 6 h (morning fast), *n* = 12–16 per group. (**F**) Serum insulin levels in HFHCD-fed ApoE KO and DKO animals (*n* = 12–16). (**G**) Intraperitoneal glucose tolerance test (IPGTT) using a fixed glucose dose of 50 mg/mouse following 6 h of fasting in ApoE KO and DKO mice fed a high-fat high-cholesterol diet for 18 weeks (*n* = 6 per group). Glucose levels were measured at 15, 30, 60, and 120 min. following glucose intraperitoneal (I.P) injection. The data are presented as mean ± SEM, * *p* < 0.05, ** *p* < 0.01, *** *p* < 0.001.

Non-alcoholic steatohepatitis has been studied by different rapidly induced animal models, but many models fail to mimic the full spectrum of human NASH. Mice that are deficient of apolipoprotein E show impaired lipoprotein clearance and when challenged with a high-fat high cholesterol diet have a rapid onset of metabolic risk factors, including steatosis, hepatic inflammation, insulin resistance, and dyslipidemia [25,32]. Previously, we used this ApoE deficient mouse model to explore the effects of Ron-dependent macrophage polarization on the development and progression of atherosclerosis and NASH [9]. Expanding on this, here we investigated the major metabolic pathways underlying NASH pathogenesis that were altered by the loss of Ron signaling. Apolipoprotein E deficient mice (ApoE KO mice) that lacked the ligand binding domain in Ron (DKO mice) were maintained on a high-fat high-cholesterol diet (HFHCD) for 18 weeks, along with age-matched control ApoE KO mice (Figure 1A). When compared to wild type C57BL/6 mice fed a high caloric diet, ApoE KO mice are more susceptible to developing states of dyslipidemia, hepatic steatosis, and steatohepatitis. Because NASH develops in parallel to adipose tissue dysfunction, type 2 diabetes, and insulin resistance in humans and other mouse models, we assessed weight gain and the morphological and molecular profiles of gonadal white adipose tissue obtained from HFHCD-fed ApoE KO and DKO mice. These mice showed no significant differences in total body weight (Figure 1B). Interestingly, DKO mice exhibited increased epididymal white adipose tissue (eWAT) mass, suggesting a possible difference in fat distribution among ApoE KO and DKO mice (Figure 1C). Furthermore, while ApoE KO control animals had restrained eWAT expansion despite the increasing total body weight, eWAT expansion in DKO mice showed a strong positive correlation to total body weight (Figure 1D). Deregulated glucose uptake and metabolism are critical consequences of unhealthy adipose tissue expansion; thus, we investigated the effects of Ron on glucose homeostasis. Although, the ApoE KO mouse model is not recognized as a susceptible model for obesity and insulin resistance, DKO mice exhibited exaggerated hyperglycemia, as higher fasting glucose levels were observed in DKO mice in comparison to ApoE KO control mice (Figure 1E). The loss of Ron also resulted in increased levels of insulin or insulinemia in HFHCD-fed mice (Figure 1F). When challenged with glucose, the DKO mice exhibited impaired insulin action or glucose tolerance (Figure 1G). Altogether, impaired Ron activation alters the metabolic state in HFHCD-fed animals resulting in unhealthy adipose tissue expansion and the deregulation of glucose metabolism.

**Figure 2:**
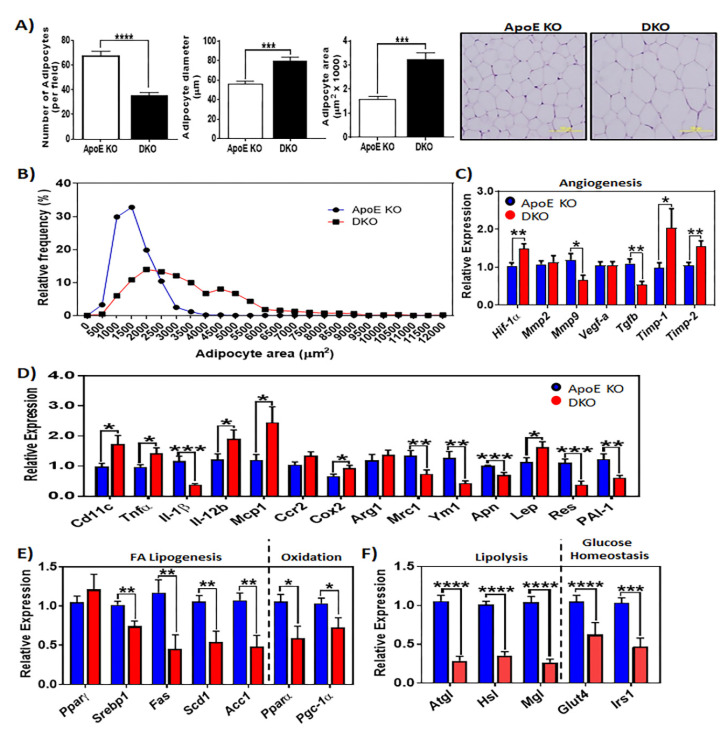
Ron receptor signaling alters the expression profile of epididymal white adipose tissue from high fat high cholesterol diet (HFHCD)-fed mice. (**A**) The number, diameter (μm), and area (μm^2^) of mature adipocytes in eWAT harvested from ApoE KO, and DKO animals (representative image shown to far right). (**B**) The frequency distribution of adipocyte area in adipose tissue from ApoE KO (*n* = 6) and DKO (*n* = 6) mice maintained on a HFHC diet. (**C**) The expression of genes that are responsive to hypoxia or mediate angiogenesis (*n* = 12–16 per group). (**D**) Quantitative RT-PCR analysis of pro-inflammatory and anti-inflammation adipokine expression in eWAT of HFHCD-fed animals. (**E**) Expression of lipogenic, fatty acid oxidation, (**F**) lipolysis and glucose uptake-related genes in epididymal white adipose tissue extracts, determined by quantitative real-time PCR (*n* = 12–16 per group). The data are presented as mean ± SEM, * *p* < 0.05, ** *p* < 0.01, *** *p* < 0.001, **** *p* < 0.0001.

We evaluated adipocyte morphology to determine the contributing factors in increased eWAT expansion in DKO mice. In expanded adipose tissue, adipocyte hyperplasia and hypertrophy are two major pathways that contribute to adipose tissue growth. Histological analysis of eWAT from DKO mice revealed extensive adipocyte enlargement or hypertrophy (Figure 2A,B). In response to adipose tissue hypertrophy, hypoxia-responsive transcriptional programs are commonly upregulated, including the expression of pro-angiogenic genes in an attempt to increase blood supply and promote tissue homeostasis. We evaluated the expression of several hypoxia-responsive genes to determine whether hypertrophic DKO eWAT exhibited these transcriptional adaptations. While DKO eWAT exhibited higher expression of hypoxia marker *Hif-1a*, pro-angiogenic genes were downregulated, as shown in Figure 2C. Although *Vegf* gene expression remained unchanged, DKO eWAT exhibited lower transcript levels of *Mmp-9*, a matrix metalloproteinase that regulates the bioavailability of VEGF and indirectly regulates angiogenesis. *Tgf-β*, a pro-angiogenic marker and regulator of *Mmp-9* expression, was also reduced in eWAT from DKO mice (Figure 2C). Additionally, eWAT from DKO mice exhibited higher expression of tissue inhibitor 1 of MMP (*Timp-1*) and tissue inhibitor 2 of MMP (*Timp-2*), which are known to limit vascularization (Figure 2C). Consistent with the unhealthy expansion of DKO eWAT, we also observed upregulated expression of pro-inflammatory markers (*CD11c*, *Tnfα*, *Il-12b*, *Cox2*, *Mcp-1*, and *Lep*) in DKO eWAT (Figure 2D). Unexpectedly, *Il-1β* was downregulated in DKO animals, inversely correlating with eWAT expansion (Figure 2D). In eWAT from DKO animals, the downregulation of anti-inflammatory markers (*Mrc/CD206*, *Ym1*, and *Apn*) further supported its deregulated metabolic state (Figure 2D). The expression of adipokines, such as plasminogen activator inhibitor-1 (*Pai-1*) and resistin (*Res*), was also decreased in the eWAT from DKO mice when compared to ApoE KO control animals (Figure 2D). These results show that the loss of the Ron receptor promotes the activation of transcriptional programs that drives WAT dysregulation.

In contrast to the enlarged adipocytes observed in the DKO mice, pro-lipogenic genes, such as *Srebp-1c* and target genes (*Fas*, *Scd1*, *Acc11*), were reduced in these cells (Figure 2E), which is a paradoxical phenomenon often associated with insulin resistance. In eWAT from DKO mice, the gene expression of fatty acid oxidation markers, *Pparα* and *Pgc-1α*, was also downregulated in these animals. Similarly, lipolytic genes encoding adipose triglyceride lipase (*Atgl*) and hormone-sensitive lipase (*Hsl*) were suppressed in DKO eWAT (Figure 2F). We assessed the transcription levels of the nutrient transport mediators glucose transporter 4, (*Glut4)* and insulin receptor substrate-1 (*Irs-1*), which are integral for normal metabolic function in WAT, in order to investigate whether glucose regulatory pathways were affected by loss of Ron. DKO WAT exhibited downregulated expression of both *Glut4* and *Irs-1* (Figure 2F), which is consistent with the impaired glucose homeostasis observed in these mice.

### 2.2. Loss of Ron Promotes Enhanced Intrahepatic Fat Storage and Increases Hepatic Fatty Acid Oxidation

**Figure 3:**
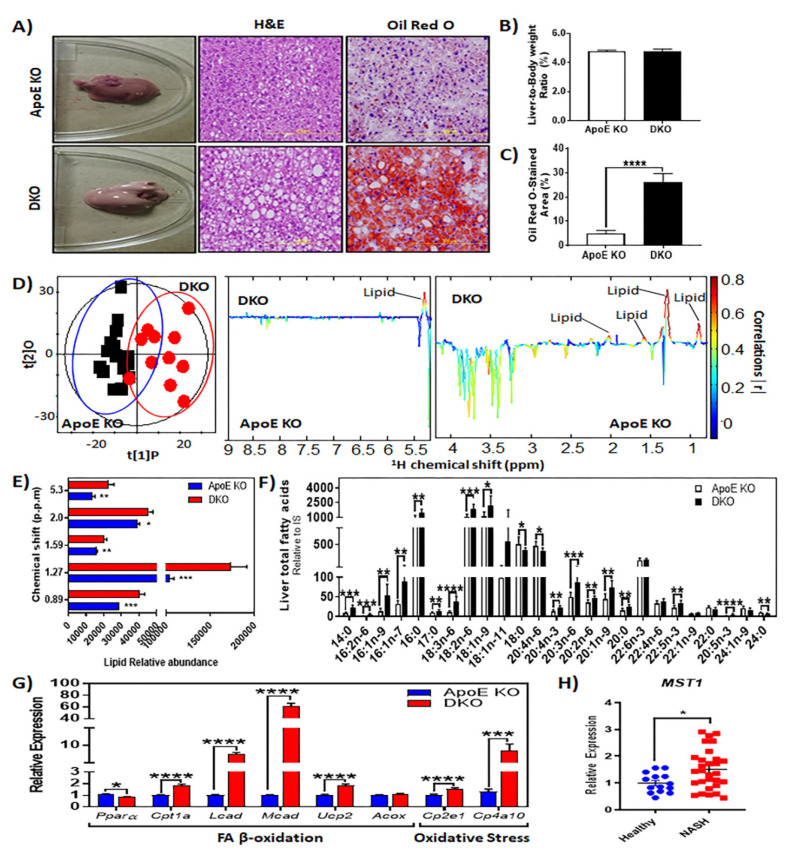
Protective role of Ron receptor signaling in steatohepatitis development and progression. (**A**) Representative images of liver gross morphology, hematoxylin and eosin (H&E) and Oil Red O (ORO) stained liver sections. (**B**) Relative liver weights of HFHCD-fed mice (*n* = 12–16 per group). (**C**) Quantitative analysis of Oil Red O stained area (%), *n* = 10 per group. (**D**) Orthogonal projection to latent structures with discriminant analysis (OPLS-DA) score plot of metabolites profiles on ^1^H NMR spectra of liver derived from HFHCD-fed mice. Each red and black circle represents one ^1^H CPMG NMR of liver of a DKO and an ApoE KO mouse, respectively. OPLS-DA coefficient plot of sera obtained from ApoE KO and DKO mice maintained on a HFHCD for 18 weeks. The upward orientation of the peaks denotes higher concentration of the metabolite in the corresponding group of HFHCD-fed mice. The color of the signals signifies the strength of relationship between the metabolite and corresponding HFHCD-fed mouse group with red representing highest significance and black representing least significance. (**E**) Quantitative representation of significant metabolites in the livers derived from ApoE KO and DKO mice. (**F**) GC-MS measured levels of fatty acids in livers extracted from HFHCD-fed animals (*n* = 8 per group). (**G**) Gene expression analysis of mitochondrial (*Cpt1a*, carnitine palmitoyltransferase; *Lcad*, long-chain acyl-CoA dehydrogenase; *Mcad*, medium-chain acyl-CoA dehydrogenase; *Ucp2*, uncoupling protein 2) and peroxisomal (*Acox*, straight-chain acyl-CoA oxidase) β-oxidation. Measured expression of genes that are involved in regulating oxidative stress including *Cyp2e1* and *Cyp4a10.* (**H**) Gene expression of macrophage stimulating protein (MSP) or MST1 in livers from healthy (*n* = 14) and NASH (*n* = 30) human patients. The data are presented as mean ± SEM, * *p* < 0.05, ** *p* < 0.01, *** *p* < 0.001, **** *p* < 0.0001.

We evaluated the phenotype of livers from ApoE KO and DKO mice to determine the influence of Ron on the onset and progression of NASH-associated steatosis. Liver weights did not differ between ApoE KO and DKO mice (Figure 3A,B). However, hematoxylin and eosin (H&E) stained cross-sections of livers revealed an exacerbated pathology in DKO animals (Figure 3A). DKO mice exhibited increased macrovesicular steatosis when compared to ApoE KO livers. which is suggestive of excessive triglyceride accumulation (Figure 3A). To further clarify whether hepatic lipid accumulation was increased in DKO livers, liver sections were stained with Oil Red O to visualize neutral lipid content (Figure 3A,C). Corroboratively, quantitative analysis of Oil Red O demonstrated that DKO livers had increased lipid storage (Figure 3C). Nuclear magnetic resonance (NMR) analysis further validated the increased lipid load in DKO livers (Figure 3D,E).

Increased triglyceride synthesis in liver is strongly linked to steatosis development and free fatty acid (FFA) is a major contributor to the intrahepatic triglyceride pool [33,34,35]. NMR analysis of liver samples derived from HFHCD-fed animals revealed significantly elevated levels of lipids in DKO livers as compared to livers of ApoE KO mice (Figure 3D,E). We then determined free fatty acid concentration and composition in livers of HFHCD-fed mice by gas chromatography mass spectrometry (GC-MS). On average, HFHCD-fed DKO mice showed elevated levels of saturated fatty acids, 14:0 (myristic acid), 16:0 (palmitic acid), 17:0 (margaric acid), and 20:0 (arachidic acid), while saturated fatty acids 18:0 (stearic acid), 22:0 (behenic acid), and 24:0 (lignoceric acid) were decreased in these livers (Figure 3F). The livers from DKO mice also had higher levels of monounsaturated fatty acids (MUFAs), such as 16:1n-7 (palmitoleic acid), 16:1n-9 (palmitoleate), 18:1n-9 (oleic acid), 20:1n-9 (gadoleic acid), and 22:1n-9 (erucic acid), while fatty acids, such as 18:1n-11 (vaccenic acid) and 22:1n-9 (erucic acid), were only moderately increased (Figure 3F). Polyunsaturated fatty acid (PUFA) levels were also elevated in livers from HFHCD-fed DKO mice with significant changes in levels of 16:2n-6, 18:3n-6 (gamma-linolenic acid), 18:2n-6 (linoleic acid), 20:4n-3 (eicosatetraenoic acid), 20:2n-6 (eicosadienoic acid), 22:5n-3 (docosapentaenoic acid), and 20:5n-3 (eicosapentaenoic acid) (Figure 3F).

We measured the expression of genes that are involved in mitochondrial (*Cpt1a*, *Ucp2*, *Lcad*, *Mcad)* and peroxisomal (*Acox*) β oxidation to determine whether increased fatty acid β oxidation contributed to the reduced lipid storage in ApoE KO (control) animals. DKO livers exhibited an increased expression of enzymes that are involved in mitochondrial fatty acid oxidation, while genes involved in peroxisomal oxidation demonstrated no difference (Figure 3G). Because oxidative stress is a major consequence of dysregulated fatty acid oxidation, we also measured the gene expression of major oxidative stress markers. In DKO mice, livers exhibited higher expression *Cyp2e1*, *Cyp4a10*, and the antioxidant enzyme catalase (Figure 3G). In our previous study, we investigated the effects of diet-induced chronic liver injury on the expression of Ron receptor ligand and mitogenic liver-derived growth factor, MSP [36]. Extensive liver injury in DKO mice was paralleled by upregulated hepatic MSP mRNA expression [36]. Similarly, this upregulation of MSP (or MST1) expression was also observed in human patients with NASH (Figure 3H).

### 2.3. Impaired Ron Receptor Signaling Results in Increased Expression of SREBP-1c and Target Lipogenic Enzymes in Livers of HFHC Diet-Fed Mice

**Figure 4:**
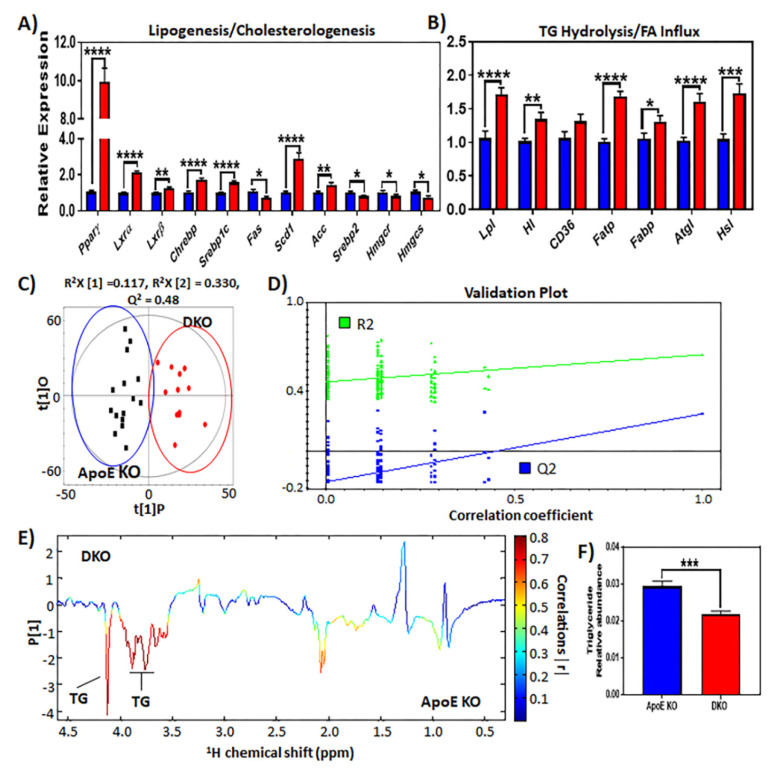
Loss of MSP-dependent Ron receptor signaling augments the hepatic lipogenic program. (**A**) Expression of hepatic genes that are involved in fatty acid, triglyceride and cholesterol synthesis in HFHCD-fed mice (*n* = 12–16 per group). (**B**) Expression of hepatic genes involved in fatty acid uptake and E. hepatic lipolysis (*n* = 12–16 per group). (**C**) OPLS-DA score plot of metabolites profiles and (**D**) validation plot based on ^1^H NMR spectra of serum samples obtained from diet-fed ApoE and DKO mice. (**E**) OPLS-DA coefficient plot on ^1^H NMR spectra of blood sera derived from ApoE KO and DKO mice maintained on a HFHCD for 18 weeks (*n* = 12–16 per group). (**F**) Quantitative representation of triglyceride levels in blood serum from ApoE KO and DKO animals. The data are presented as mean ± SEM, * *p* < 0.05, ** *p* < 0.01, *** *p* < 0.001, **** *p* < 0.0001.

We investigated the transcriptional regulation of major FFA biosynthesis pathways, such as sterol regulatory element binding-1c (SREBP-1c) and carbohydrate-responsive element-binding protein (ChREBP), in order to determine whether deregulated de novo lipogenesis contributed to the increased FFA pool in DKO livers. Livers from DKO mice exhibited increased expression of *Srebp-1c*, *Chrebp*, and downstream targets stearoyl-CoA desaturase-1 (*Scd1*) and acetyl-CoA carboxylase (*Acc1*), whereas fatty acid synthase (*Fas*) expression was reduced (Figure 4A). Interestingly, the expression of cholesterogenic transcription factor *Srebp2* and downstream targets *Hmgcr* and *Hmgcs* was downregulated in HFHCD-fed DKO mice when compared ApoE KO mice (Figure 4A).

The expression of peroxisome proliferator-activated receptor gamma (PPARγ), which is known to regulate hepatic de novo lipogenesis (DNL) and free fatty acid (FFA) uptake, was upregulated in DKO livers (Figure 4A). Additionally, the expression of fatty acid transport protein-1 (*Fatp-1*/*Slc27a1*) and liver-type fatty acid binding protein (*L-Fabp)* were also enhanced in livers from DKO animals (Figure 4B). Lipoprotein lipase (LPL) and hepatic lipase (HL) are key enzymes that mediate hydrolysis and the clearance of triglyceride and phospholipids in circulation. In DKO livers, the expression of genes encoding these enzymes was upregulated in comparison to control ApoE KO animals (Figure 4B) and, thus, may have contributed to the lowered serum triglyceride levels also observed in these animals (Figure 4C–F). Additionally, the expression of lipolytic enzymes involved in triglyceride catabolism (*Hsl* and *Atgl*) was increased in livers from DKO mice (Figure 4B). Together, our results suggest that the upregulated transcription genes positively regulating *de novo* lipogenesis fatty acid synthesis, fatty acid uptake, and intrahepatic triglyceride catabolism led to the increased fatty acid storage in DKO livers.

### 2.4. Ron Receptor Signaling Affects Bile Acid Synthesis and Metabolism in HFHC-Diet-Fed Mice

**Figure 5:**
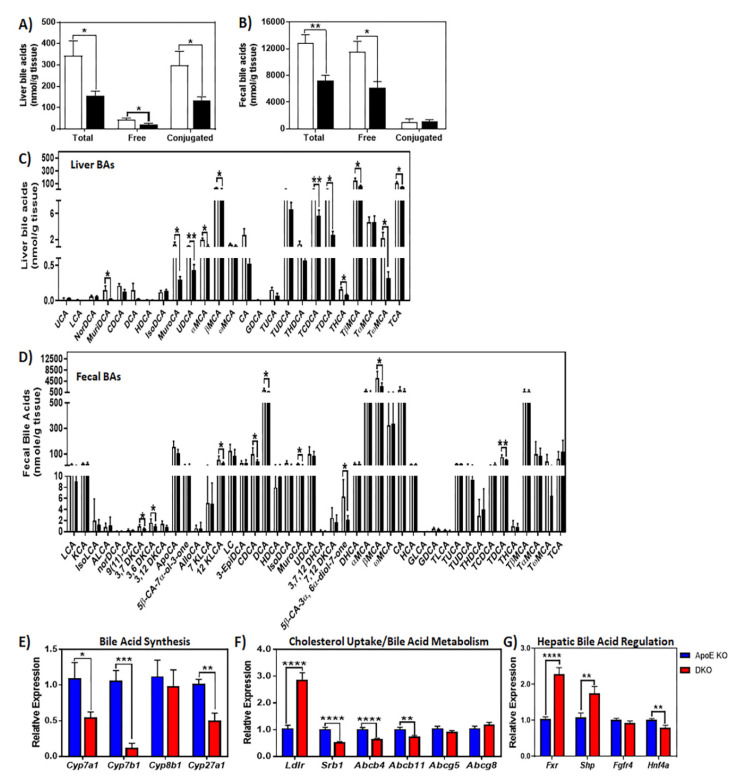
Impaired Ron receptor signaling alters bile acid synthesis and metabolism. (**A**,**C**) Bile acid concentrations in liver and (**B**,**D**) feces from mice fed HFHCD for 18 weeks (*n* = six per group). The data are presented as mean ± SEM, * *p* < 0.05, ** *p* < 0.01, *** *p* < 0.001, **** *p* < 0. 0001. Quantitative RT-PCR analysis of genes involved in (**E**) bile acid synthesis (*n* = five per group), (**F**) cholesterol uptake/bile acid metabolism regulatory and (**G**) bile acid regulation livers of HFHCD-fed mice (*n* = 12–16 per group).

Another route for lipid disposal in the liver is through cholesterol catabolism, bile acid synthesis, and biliary cholesterol secretion. Targeted LC/MS was used to measure the concentration of bile acids in multiple tissues derived from diet-fed mice. Hepatic and fecal bile acids were decreased in DKO mice when compared to ApoE KO animals (Figure 5A–D; Supplementary Table S1). Although not as significant, this trend was also observed with circulating bile acids in DKO mice (Supplementary Figure S1; Supplementary Table S1). In support of these findings, livers from DKO animals exhibited decreased expression of classical and alternative bile acid synthesis enzymes (Figure 5E). The expression level of *Srb1*, which encodes the scavenger receptor, class B type 1 (SR-B1), a receptor for biliary high-density lipoprotein (HDL)-cholesterol, was decreased in livers of DKO mice (Figure 5F). On the other hand, the gene encoding low density lipoprotein receptor (LDLR), which is responsible for the uptake of the non-preferred sterols in biliary secretion, was significantly elevated in diet fed ApoE KO mice lacking normal Ron receptor signaling (Figure 5F). Additionally, the expression of genes encoding hepatic transporters regulating the secretion of bile acids (ATP binding cassette subfamily B member 11, *Abcb11*) and other bile constituents such as phospholipids (ATP binding cassette subfamily B member 4, *Abcb4*) was decreased in DKO livers (Figure 5F). However, the expression of biliary cholesterol transporters such as ATP binding cassette subfamily G member 5/8 (*Abcg5/8*) showed no difference between groups (Figure 5F). We next evaluated transcription factors that are known to suppress genes required for bile acid synthesis. A drastic increase in *Fxr* (farnesoid x receptor) expression was observed in DKO mice, as well as for its immediate downstream nuclear factor, small heterodimer partner or *Shp* (Figure 5G). There was no change observed in fibroblast growth factor receptor 4 (*Fgfr4*) expression (Figure 5G). Consistent with the decreased expression of gene encoding enzymes required for bile acid synthesis, decreased hepatocyte nuclear factor 4 alpha (*Hnf4a*) expression was also observed in DKO mice (Figure 5G).

## 3. Discussion

**Figure 6:**
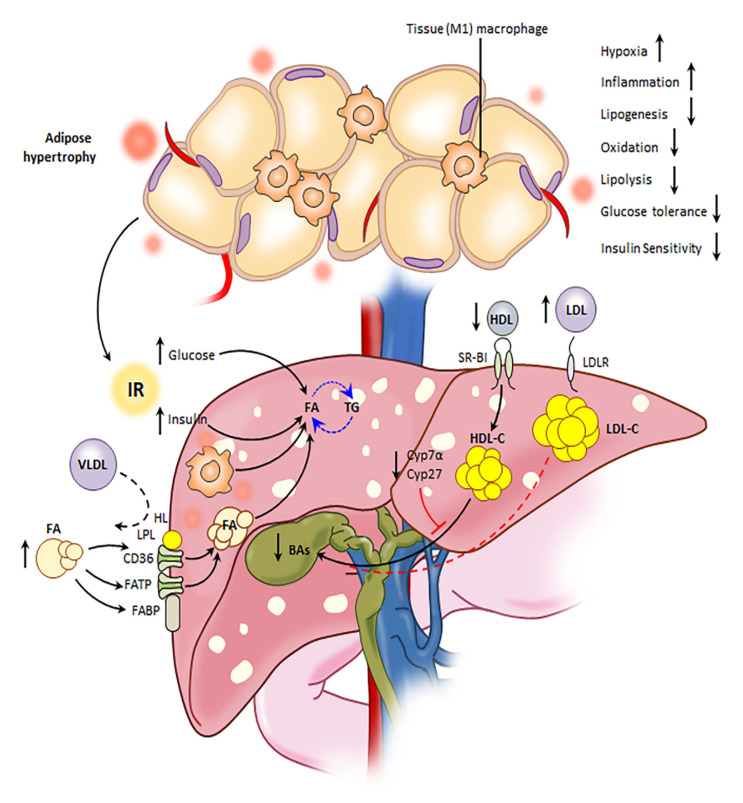
Schematic summary of the metabolic outcome of impaired Ron receptor signaling.

Non-alcoholic steatohepatitis (NASH) is a major health burden that is associated with the metabolic syndrome. Imbalances in hepatic lipid storage and mobilization pathways can lead to steatosis, inflammation, and oxidative stress, all key characteristics of NASH. These insults can disrupt liver homeostasis and cause irreversible scarring of the liver or cirrhosis. Our current study demonstrates the effects of Ron receptor signaling on major lipid metabolism pathways that are involved in the initiation and aggravation of steatohepatitis in vivo (Figure 6). We demonstrated that white adipose tissue homeostasis and insulin sensitivity was deregulated in DKO animals, which could partially contribute to the exacerbated steatohepatitis observed in these animals. Despite extensive expansion of eWAT, lipogenic markers were markedly downregulated. Several reports offer explanations for this paradoxical phenomenon often present in obesity and insulin resistance [37,38,39]. *Srebp-1c* expression has been shown to be directly regulated by insulin in the 3T3-L1 adipocyte-like cell line, mice, and humans [37,38,40,41]. Because IRS-1 is critical for insulin receptor responsiveness [42,43], the downregulated expression of *Irs-1* in the eWAT of DKO mice may render insulin incapable or exerting its positive regulatory effects on SREBP-1c.

Oxygen deprivation in hypertrophic adipose tissue triggers the expression of hypoxia, angiogenic, and inflammatory markers [44,45]. Along those lines, the enlarged adipocytes of DKO WAT may act to compromise effective oxygen supply from the vasculature and upregulate *Hif-1α* gene expression. Although the upregulation of pro-angiogenic markers is a common adaptive response to tissue hypoxia [44,46,47], DKO WAT exhibited downregulated pro-angiogenic gene expression (*Mmp-9* and *Tgfβ*), whereas anti-angiogenic markers (*Timp-1* and *Timp-2*) were increased. Altogether, hypertrophy and suppressed pro-angiogenic transcriptional programs may both act to regulate genes, such as *Hif-1α*. The exaggerated WAT dysfunction in DKO mice was further supported by imbalances in the expression of inflammatory and anti-inflammatory mediators. We have previously shown the effects of Ron in a diet-induced obesity (DIO) model [9]. In the DIO model, wild-type (WT) and Ron knockout (KO) mice on a C57BL/6 background were maintained on a high-fat diet. In WT mice maintained on an HFD, we show that the expansion of visceral fat is negatively correlated to Ron expression on adipose tissue-resident macrophages (ATMs). Ron KO mice showed increased body weight and exhibited increased recruitment of CD11c-positive M1-like macrophages in their visceral fat when compared to WT mice. Ron surface expression on CD11c-positive and CD11c-negative ATM populations that were isolated from WT mice was evaluated. CD11c-positive M1-like ATMs exhibited lower expression of Ron in comparison to CD11c-negative M2-like ATMs. CD11c^+^ M1 ATMs is well reported as a major source of inflammatory cytokines in adipose tissue [9]. Similarly, in our present findings using a NASH ApoE-deficient mouse model, transcriptional profiling of eWAT isolated from ApoE KO and DKO mice showed a differential expression of CD11c. DKO-derived eWAT exhibited significantly higher expression of CD11c, a well-known and reported M1 macrophage marker. In further support of these findings, proinflammatory mediators that are strongly associated with a M1-like macrophage phenotype, including *Tnfα*, *Il-12b*, *Mcp1*, and *Cox2*, showed higher expression in DKO-derived eWAT. On the other hand, ApoE KO-derived eWAT exhibited higher expression of well-reported macrophage-specific anti-inflammatory markers, *Mrc1* and *Ym1*. Adiponectin is a fat-derived hormone that is known to have protective actions against the initiation and progression of insulin resistance and atherosclerosis. Reduction of this adipocytokine plays a central role in the pathogenesis of these obesity-related diseases. DKO-derived WAT exhibited a decreased expression of adiponectin (*Apn*). Leptin is also an adipocytokine synthesized and secreted specifically by WAT and has peripheral actions that contribute to type 2 diabetes, insulin resistance, and atherosclerosis. In DKO-derived eWAT, the expression of leptin (*Lep*) was higher than that of ApoE-derived eWAT.

Previously we have shown that the loss of Ron activity in mice results in elevated serum FFA levels, another marker for AT expansion and dysregulation [36]. In contrast, pro-lipolytic lipase genes in DKO eWAT were downregulated, which may be due to the inhibitory effects of hyperinsulinemia on adipocyte-specific *Atgl* and *Hsl* gene expression [48,49,50]. Furthermore, the increased fat stores in DKO adipocytes could be the outcome of suppressed lipase-mediated TG mobilization following AT inflammation and insulin resistance. In DKO animals, the elevation in insulin desensitizing factors, such as FFAs and cytokines, is believed to contribute to the deregulated glucose metabolism. In support of this, we show that these mice exhibited downregulated *Irs-1* and *Glut4* expression, hyperglycemia, insulinemia, and impaired glucose clearance. Altogether, DKO mice displayed reduced glucose tolerance and insulin sensitivity, which are established risk factors of insulin resistance (IR) and type 2 diabetes. In other reports, the Met receptor, which is closely related to Ron, has also been shown to improve insulin resistance. In vitro activation of Met on adipocytes stimulated glucose uptake through the increased translocation of Glut4 but not Glut1 [51,52,53].

In addition to AT dysregulation, we show that loss of Ron exacerbated hepatic steatosis through the upregulation of major pro-lipogenic pathways such as SREBP-1c and ChREBP. We speculate that the increased levels of pro-lipogenic factors, insulin, and glucose in DKO animals contributed to the exaggerated steatosis by activating highly responsive transcription factors, SREBP-1c and ChREBP. Of note, Ron is abundantly expressed on hepatocytes and, thus, we cannot rule out the possibility that these lipogenic programs are directly regulated by Ron receptor activation in hepatocytes. A recent study showed that ex vivo stimulation of the hepatocyte-specific Ron receptor downregulates SREBP-1c encoding gene, *Srebp-1c* and its target genes [54]. This is consistent with the increase in hepatic *Srebp-1c* gene expression and SREBP-1c downstream target genes in DKO mice. Because inflammatory stress can induce hepatic lipogenesis [55,56,57], the exacerbated inflammation in DKO livers may also facilitate lipid accumulation in liver. Overall, hyperinsulinemia and inflammation in DKO mice may both synergistically trigger steatosis by activating major *de novo* lipogenesis pathways.

A growing body of literature supports that insulin induces SREBP-1c activation by regulating LXRα [58,59,60,61]. Insulin acts primarily by increasing LXRα activation of SREBP-1c promoter [58,59,61]. In support of this, livers of DKO mice showed increased expression for a majority of LXRα target genes, including *Srebp-1c*, *Chrebp*, *Abca1*, *Lpl*, and *Lxrα*. Further support for LXRα activation includes its inhibitory actions on cholesterol biosynthesis genes. In other studies, LXRα null mice were demonstrated to have higher expression of *Srebp-2*, as well as downstream responsive genes *Hgmcs* and *Hmgcr* [62,63]. In agreement with this, hepatic expression of *Srebp-2*, *Hgmcr* and *Hmgcs* was downregulated in DKO animals. Despite the decreased expression of Srebp-2 responsive genes, low-density lipoprotein receptor (*Ldlr*) expression was upregulated in livers of DKO animals. These results suggest that *Ldlr* RNA expression is regulated by a mechanism independent of the SREBP-2 activation. A possible explanation for the upregulated *Ldlr* expression is its regulation by insulin-induced SREBP-1 activation. Insulin can increase *Ldlr* expression in hepatocyte cell lines through the recruitment of SREBP-1c to its sensitive cis sterol regulatory element 1 (SRE1) in the LDLR promotor [64,65]. Furthermore, our results points to the possibility that increased *Ldlr* expression may favor the uptake of cholesterol-rich LDL and, thus, act to maintain large lipid pools in DKO livers.

Another contributing source for hepatic fat accumulation is the increased influx of free fatty acid uptake into the liver. Hydrolysis of plasma TG/phospholipid and fatty acid uptake is a process regulated, in part, by PPARγ and its downstream targets, CD36, HL, LPL, FATP, and FABP [66,67,68,69,70,71]. The upregulation of the PPARγ-encoding gene *(Pparγ*) and PPARγ responsive genes, as well as the depleted circulating levels of TG and phospholipid in DKO mice suggest that fatty acid uptake by the liver is a crucial contributing factor in the increased fat accumulation in these livers. Hepatic lipase and lipoprotein lipase mediate the hydrolysis of circulating phospholipids and triglycerides and the release of free fatty acids facilitating FFA influx into the liver. The overexpression of these lipases in mice has been shown to lower plasma TG [72,73,74]. Metabolomic profiling, including NMR and GC-MS analysis, revealed that livers of DKO mice exhibited higher concentrations of saturated and polyunsaturated fatty acids. Polyunsaturated fatty acids, specifically n-3 or n-6 PUFAs, are natural agonists of PPARγ [75,76].

Triglyceride catabolism and fatty acid oxidation are common adaptive responses to steatosis [77]. Not surprisingly, the maladaptive upregulation of genes encoding the adipose triglyceride lipase (ATGL) and hormone sensitive lipase (HSL) was observed in DKO livers. The upregulation of transcriptional programs in fatty acid oxidation and oxidative stress in DKO mice further suggests the contributing factors accelerating steatohepatitis in these animals. Another route for lipid disposal, which includes cholesterol catabolism or bile acid synthesis, was suppressed in the absence of Ron signaling. In diet-fed animals, loss of Ron downregulated genes encoding bile acid synthesis enzymes (*Cyp7a1*, *Cyp7b1*, and *Cyp27a1*) and that are crucial for BA metabolism (*Srb1*, *Abcb4*, and *Abcb11*). It is noteworthy that in bile acid synthesis and secretion, HDL-cholesterol is preferentially used and trafficked into the liver exclusively by SR-B1 [78,79,80,81,82,83,84,85]. Our results suggest that, in DKO livers, increased *Ldlr* and decreased *Srb1* levels contribute to unfavorable imbalances in non-HDL and HDL-cholesterol pools, and thus contribute to the decreased bile acid synthesis and bile acid concentrations observed. Furthermore, this suggests that cholesterol mobilization in ApoE KO and DKO livers is mediated by differential partitioning of non-HDL- and HDL-cholesterol pools. Increased uptake of low-density lipoprotein (LDL) cholesterol by highly expressed LDL receptor (LDLR) in concert with lowered expression of high-density lipoprotein (HDL) receptor SR-BI, is believed to limit the availability of preferred catabolic substrate, HDL-cholesterol for bile acid synthesis.

Other possible contributing factors underlying the DKO bile acid profile involves the well-known inhibitory actions of hepatic FXR/SHP signaling axis on the hepatic CYP7A1 upstream regulator, HNF4A1 [86,87]. Insulin has been shown to negatively regulate *Cyp7a1* and *Cyp27a1* expression and, thus, bile acid synthesis [86,88,89]. Therefore, hyperinsulinemia in DKO animals may play a role in suppressing bile acid synthesis in the liver. The HNF4A1/CYP7A1 pathway is also suppressed by cytokines that are produced by Kupffer cells. We have previously shown that the livers of DKO mice exhibit a higher expression of macrophage-specific inflammatory mediators including *Il-12b*, *Tnfα*, and *Inos* [9,36]. The decreased synthesis of bile acids in DKO-derived liver may be in response to the exacerbated inflammatory response induced primarily by classically activated (M1) liver macrophages. In other studies, the depletion of resident macrophages using clondronate-containing liposomes have supported the well-reported involvement of Kupffer cells in the development and progression of NASH, with proinflammatory macrophages determining the disease severity [16,90,91,92]. Along those lines, in a prior study, we showed that DKO mice exhibited increased macrophage recruitment and inflammation that, in turn, accelerated the onset of fibrosis [36]. Kupffer cells are first responders to hepatic injury and the production of inflammatory cytokines, such as TNFα, act to propagate hepatocellular insults, initiate inflammation, and accelerate NASH. In addition to danger molecules that are released by damaged hepatocytes, Kupffer cells are also classically activated by a flux of circulating free fatty acids and adipose tissue insulin resistance into the liver [93,94,95]. In humans, the classical activation of hepatic macrophages is paralleled by both circulating level of FFAs and adipose tissue insulin resistance [96]. The favored M1 polarization of Kupffer cells by FFAs overflow is mediated through the toll like receptor-4 pathway [97,98,99]. We have previously demonstrated that MSP-induced Ron signaling can suppress TLR-4 signaling in primary macrophages thereby inhibiting a classically activated (M1) response [11]. TLR-4 signals to induce IKK-mediated phosphorylation of IκB, which is followed by its proteasomal degradation and the subsequent translocation of NF-κB. Stimulation of primary macrophages with MSP delays IKK activity and IκB degradation, which limits serine phosphorylation of p65 and reduces NF-KB transcriptional activity. Altogether, the increased FFA pool in DKO livers may serve as a major stimulus for Kupffer cell activation. Consequently, the lack of normal Ron signaling and resulting diminished anti-inflammatory responses in DKO Kupffer cells would further aggravate TLR4-mediated macrophage activation. The unsuppressed classical activation of Kupffer cells act to establish and maintain chronic low-grade inflammation in DKO livers, exacerbating the development of NASH in these animals.

Chronic liver injury is a well-recognized for stimulating increased expression of liver-derived growth factor, including MSP (or hepatocyte growth factor like; HGFL) and structurally related protein hepatocyte growth factor (HGF) [100,101,102]. MSP/HGFL and HGF both function to promote liver differentiation, regeneration, and tissue repair. Both Ron and the closely-related Met receptor are protective in hepatic fibrosis [36,103,104,105,106]. Recombinant HGF, the ligand for Met, suppressed the progression in a mouse model of NASH, and MSP negatively regulated inflammation and lipogenesis in ex vivo models of NASH [54,106,107]. Ron and Met primarily signal through the large adaptor protein, Gab1 and loss of Gab1 has been shown to aggravate experimental liver fibrosis in mice [108,109]. Furthermore, both Ron and Gab1 are protective in models of acetaminophen-induced liver injury in mice. A growing body of literature points to HGF as a potential marker and treatment for liver injury [110,111,112,113,114]; however, similar potential has not been extended to MSP/HGFL. Our current findings spotlight the biomarker and therapeutic potential of the MSP in NASH.

## 4. Materials and Methods

### 4.1. Animal Model and Diet

Six-week-old male apolipoprotein E deficient (ApoE KO) mice and Ron receptor tyrosine kinase deficient ApoE KO mice (DKO) were maintained on a high fat high cholesterol diet (HFHCD) for 18 weeks. The high fat high cholesterol diet consisted of 60% fat calories and 1.25% cholesterol and it was purchased from Bio-Serv (diet number F6334; Flemington, NJ, USA). DKO mice were generated, as previously described [9]. All of the mice were maintained or generated on a C57BL/6 background. The animals were housed in a temperature-controlled room on a 12-h light/dark cycle. Experimental protocols used throughout this study were approved by the Pennsylvania State University Institutional Animal Care and Use Committee, PRAMS 200946345 (17 January 2020). The mice were euthanized by CO_2_ asphyxiation.

### 4.2. Preparation of Serum, Feces and Tissue

Blood was isolated from euthanized mice using cardiac puncture. Serum was separated from whole blood by centrifugation and frozen at −80 °C until further use. Livers were rapidly excised, weighed, and aliquots were stored accordingly. The liver aliquots were either snap-frozen in liquid nitrogen or stored at −80 °C for western blotting analysis, real-time polymerase chain reaction (PCR), bile acid analysis by liquid chromatography-mass spectrometry (LC-MS), or fatty acid analysis by gas chromatography-mass spectrometry (GC-MS). Additional liver aliquots were fixed in 10% formalin or frozen in optimum cutting temperature (OCT) media for histology. Epididymal fat pads were excised, weighed, snap-frozen in liquid nitrogen, and stored at −80 °C for RT-PCR analysis. Feces were rapidly removed from the resected colon, snap-frozen in liquid nitrogen and stored at −80 °C for bile acid analysis by UPLC-MS.

### 4.3. Glucose Homeostasis Assessment

Mice were housed in cages with woodchip bedding and deprived of food for 5–6 h. A OneTouch Ultra blood glucose monitoring system (LifeScan; Wayne, PA, USA) was used to measure glucose levels in blood that was acquired from the tip of the tail. Glucose tolerance tests were performed on age matched mice fasted 5–6 h (morning fast). A fixed glucose solution (50 µg) was administered via intraperitoneal injection (I.P). The glucose levels were measured through the acquisition of blood sample from the tail tip at indicated time points. Insulin levels were measured using the ultra-sensitive mouse insulin ELISA kit according to manufacturer’s instructions (Crystal Chem Inc; Elk Grove Village, IL, USA).

### 4.4. Adipocyte Histomorphometry

Epidydimal white adipose tissue was excised, washed, and fixed in 10% Neutral formalin for 48 h. Following paraffin embedding and sectioning (10 μm), adipose tissues were stained with hematoxylin and eosin (H&E). To determine adipocyte size and number per frame, the images of H&E stained sections were captured at 20× magnification and analyzed using Image J software. To quantify the number of adipocytes, adipocytes were counted under 20× magnification; a minimum of four consecutive microscopic fields per animal were evaluated for six mice per group. A minimum of 100 adipocytes per animal were manually measured using Image J in order to determine adipocyte diameter (μm) and area (μm^2^). Adipocytes that were touching the border of the field were excluded. The frequency distribution of adipocyte size was accomplished using Graph Pad Prism 7 (San Diego, CA, USA). Representative images for adipocyte size were captured at 20× magnification (scale bar represents 100 μm).

### 4.5. Sample Preparation and ^1^H Nuclear Magnetic Resonance Spectroscopy

200 µL of serum was mixed with 400 µL of saline solution containing 50% D_2_O. Liver (50 mg) were extracted two times with precooled methanol-water mixture (2/1, *v/v*) using a PreCellys Tissue Homogenizer (Bertin Technologies; Rockville, MD, USA). Following centrifugation at 11,180× *g* for 10 min. at 4 °C, the supernatants were dried using a Eppendorf Vacufage vacuum concentrator. The dried aqueous extracts were reconstituted with 600 μL phosphate buffer containing 50% D_2_O and 0.005% 3-(trimethylsilyl) (2,2,3,3-^2^H_4_) propionate (TSP-d4) (chemical shift reference). The reconstituted aqueous extracts were centrifuged at 11,180× *g* for 10 min. at 4 °C. For prepared serum and liver samples, 550 µL of the supernatant was transferred to 5 mm NMR tubes. At ambient temperatures of 298 K, a Bruker Avance III 600 MHz spectrometer armed with Bruker inverse cryogenic probe (Bruker Biospin, Rheinstetten, Germany) was employed in order to obtain the ^1^H spectra of serum and liver. A typical one-dimensional NMR spectrum for liver samples was acquired using the first increment of nuclear overhauser effect spectroscopy sequence with presaturation (NOESYPR1D). For serum, the water-presaturated Carr-Purcell-Meiboom-Gill (CPMG) pulse sequence and diffusion-edited spectra were employed to obtain and low molecular weight metabolites and macromolecules such as lipids, lipoproteins, and long-chain fatty acids, separately. For each sample, the 90° pulse width was set to approximately 10 μs. For each spectrum, 64 transients were collected into 32K data points with a spectral width of 20 ppm. A range of 2D NMR spectra including ^1^H–^1^H correlation spectroscopy (COSY), ^1^H–^1^H total correlation spectroscopy (TOCSY), ^1^H–^13^C heteronuclear single quantum correlation (HSQC), and ^1^H–^13^C heteronuclear multiple bond correlation spectra (HMBC) were acquired and processed for selected samples in order to facilitate the NMR signal assignment process.

An exponential function with a 1.0 Hz line broadening factor was multiplied with all free induction decays (FID) before the Fourier transformation. The spectra were referenced to TSP-d_4_ at *δ* 0.00 for liver and the anomeric proton signal of α-glucose at *δ* 5.23 for serum. The AMIX software package (V3.8, Bruker-Biospin) was used to integrate the spectral region *δ* 0.50–9.50 into regions with an equal width of 0.004 ppm (2.4 Hz). The region δ 4.60–5.15 for imperfect water saturation was removed. Individual ^1^H bucketed spectra regions was normalized to the total sum of spectral integrals to compensate for the differences in concentration.

The SIMCA-P+ software (version 13.0, Umetrics, Sweden) was used to perform multi-variate data analysis. The NMR data were subjected to principal component analysis (PCA) and orthogonal projection to latent structures with discriminant analysis (OPLS-DA). In order to ensure reliability of the OPLS-DA model, it was validated through a seven-fold cross validation method. The goodness-of-fit parameters for the OPLS-DA model included R^2^X and Q^2^ values. The fraction of variance of the x and y variable explained by the OPLS-DA model was represented by parameters R^2^X values. The predictive performance of the model was represented by Q^2^. Back transformed loadings generated from the OPLS-DA were plotted against ^1^H chemical shift with critical value of coefficients (|r|) as color codes. MATLAB (The Mathworks, Inc.; Natick, MA, USA) was used to generate color-coded correlation coefficient loading plots. The color-coded correlation loading plots showed the significance of the metabolite contribution to the class separation. The “hot” color (e.g., red) represented the highest significance and the “cold” color (e.g., blue) represented the least significance. In this study, the Pearson correlation coefficient critical value |r| > 0.533 (r > +0.533 and r < −0.533) was used to determine significance at a *p*-value threshold of 0.05.

To quantify the abundance of key metabolites in liver samples, each spectrum was corrected for phase- and baseline-distortions manually with the chemical shift referenced to TSP (δ 0.00). The spectral region at δ 0.5−10.0 was then integrated into bins of 0.004 ppm while using AMIX package. The regions at δ4.20−5.20 were discarded to eliminate the effects of imperfect water suppression. The areas of all bins were then normalized to the total intensity. The relative content of metabolites was calculated using the values derived from normalizing the NMR peak area normalized to total integration for each sample. The relative contents of key liver metabolites are represented as mean ± SEM. Statistical significance was determined by performing an unpaired two-tailed Student *t* test set a threshold of 0.05.

### 4.6. Liver Histopathological Analysis

Following euthanasia, the livers were excised and either fixed in 10% neutral buffered formalin or embedded in OCT. Hematoxylin & Eosin (H&E) and Oil Red O (ORO) staining were performed on fixed paraffin or OCT embedded livers, respectively, by Histoserv, Inc (Gaithersburg, MD, USA). Images of stained liver sections were captured on bright field microscope at a magnification of 20×. Representative images of liver cross-sections were captured under 20× magnification (scale bar = 100 μm). A minimum of four frames per animal were captured. For quantitative analysis of Oil Red O staining, the ORO positive area was measured using ImageJ and the average ORO positive area per captured frames for an individual animal was calculated.

### 4.7. RT-PCR Analysis

Whole epididymal white adipose tissue and liver were pulverized by a homogenizer in RiboZol RNA Extraction Reagent (VWR; Radnor, PA, USA), as instructed by the manufacturer. RNA was quantified using a Nanodrop spectrometer at an absorbance of A260. cDNA was synthesized from 2 μg of RNA using the High Capacity Reverse Transcription Kit (Applied Biosystems; Foster City, CA, USA). The generated cDNA was assessed for gene expression while using FAM-labeled Taqman probes (Applied Biosystems; Foster City, CA, USA). Real time PCR was performed on the 7900HT Fast Real-Time PCR System (Applied Biosystems; Foster City, CA, USA). Fam-labeled Taqman probes were purchased from (Applied Biosystems; Foster City, CA, USA). Threshold cycle values were normalized to the housekeeping gene *Gapdh*.

### 4.8. GC-MS Analysis of Fatty Acids

Fatty acid concentrations in liver were assessed by gas-chromatography mass spectrometry, as previously described [115]. 30 mg of liver tissue was thoroughly homogenized in a methanol: chloroform (2:1) mixture containing 50 µM of fatty acid internal standards, pentadecanoic acid (C15:0 fatty acid), and C_11_ vaccenate methyl ester. 0.9% saline was added to the supernatants and centrifuged (20,800× *g*, 15 min, 4 °C). The organic phase was dried down under N_2_. Methanolic HCL was added to the dried samples and incubated overnight at 60 °C. Hexane and 0.9% saline were added to the samples, sonicated, and the hexane fraction was dried down under N_2_. Hexane was added to the dried samples and the samples were transferred to crimp vials for GC-MS analysis. On an Agilent 7890A-5975C GC−MS system (Agilent Technologies; Santa Clara, CA, USA) equipped with a HP-5MS capillary column (30 m, 0.25 mm ID, 0.25 μm film thickness), fatty acid composition was measured. At a flow rate of mL/min., helium was employed as the carrier gas. The sample injection volume was 0.5 mL under 10 psi pressure maintained at pulsed split ratio of 1:10. The column temperature was regulated, as follows, the initial temperature was held at 80 °C for 1 min. Column temperature was gradually increased from 80 °C to 160 °C (at a rate of 20 °C/min), then to 220 °C (at a rate of 2 °C/min), followed by an increase to 310 °C (at a rate of 15 °C/min), which was held for 2 min. Normalized integrated peak areas were compared to internal standards to quantify fatty acids.

### 4.9. UPLC-MS Analysis of Bile Acids

Liver tissue and fecal samples (50 mg) were homogenized in 500 µL pre-cooled methanol containing deuterated internal standards (5 µM in H_2_O/acetonitrile/2-propanol; Supplementary Table S1) using the Precellys tissue homogenizer (Bertin Technologies; Rockville MD). After repeated extraction, samples were incubated at −20 °C and then centrifuged (11,200× *g*, 15 min, 4 °C). Supernatants were transferred to crimp vials for liquid chromatography/mass spectrometry (LC-MS) analysis. For targeted bile acid profiling, a Xevo TQ-S mass spectrometer was coupled to an ACQUITY ultraperformance chromatography (UPLC) system (all from Waters, Milford, MA, USA). Both mass spectrometer systems utilized an electrospray ionization service operating in negative ion mode (ESI-). The reversed phase chromatographic methods were performed using an AQUITY BEH 8 Column (1.7 μm, 100 mm, 2.1 mm) at 60 °C. Solvents A (9% acetonitrile in ultrapure water, 1mM ammonium acetate, acetic acid adjusted pH of 4.15) and B (1:1 *v/v* mixture of acetonitrile/2-proponol) were used in the liquid mobile phase for gradient separation of bile acids, as previously described [116]. The injection volume for sample analysis was 5 ul. Wash cycles of three were performed concurrently with sample analysis using wash (2-propanol) and purge (10% 2-propanol) solvents to minimize injection carryover and sample contamination. Mass spectrometry was performed under adapted conditions, as previously described [116]. Capillary and cone voltage were set at 1.9 kV and 60 V, respectively, at a source temperature of 150 °C and a desolvation temperature of 600 °C. The desolvation gas flow was set at 1000 L/h, while the cone gas flow was set to 150 L/h. Multiple reaction monitoring (MRM) or selected ion monitoring (SIM) was used to evaluate bile acid species yielding characteristics that are based on their fragmentation.

### 4.10. Human Liver Gene Expression Analysis

As previously described [117], human liver samples that were derived from healthy (control) and NASH patients were obtained from the Liver Tissue Cell Distribution System. Human subject data were obtained from studies conducted according to the criteria set by the Declaration of Helsinki principles that were approved by the Institutional Review Boards.

The samples were homogenized in TRizol Reagent, as instructed by the manufacturer. cDNA was synthesized from 1 μg of RNA using the 1st strand cDNA Synthesis System for Quantitative RT-PCR (OriGene; Rockville, MD, USA). The generated cDNA was assessed for gene expression using FAM-labeled Taqman probes (Applied Biosystems; Foster City, CA, USA).

### 4.11. Statistics

The data values are represented as mean ± SEM. Statistical significance was determined by performing an unpaired two-tailed Student *t* test set a threshold of 0.05. Significant differences are represented as * *p* < 0.05, ** *p* < 0.01, *** *p* < 0.001, ****p < 0.0001). All of the analyses were performed using GraphPad Prism 7.0 (San Diego, CA, USA)

## 5. Conclusions

Here, we show, for the first time, the consequences of impaired MSP-mediated Ron activation on metabolic pathways that are responsible for triggering and sustaining NASH. The loss of MSP responsiveness by the mutant form of Ron present in DKO mice accelerated non-alcoholic steatohepatitis by facilitating the onset of multiple parallel insults on the liver including WAT dysfunction, enhanced de novo lipogenesis, inflammation/oxidative stress, and reduced cholesterol mobilization (Figure 6). A limitation of this study is the use of a whole-body Ron knockout. However, by understanding the main pathways that are affected by Ron signaling, we can next investigate the role of Ron on different cell types—known to regulate these pathways using in vitro methods and in vivo cell-specific Ron KO murine models. This will bring us closer to determining whether manipulating Ron receptor signaling can be an effective therapeutic strategy for not only treating steatohepatitis, but other obesity-associated disorders.

## Supplementary Materials

The following are available online at https://www.mdpi.com/2218-1989/10/8/326/s1, Table S1: List of liver, fecal, serumbile acid, and internal standards (IS) names and abbreviations, Figure S1: GC-MS analysis of bile acid concentration in serum derived from HFHCD-fed ApoE KO and DKO mice.
