# Supplementary material for: Metabolic Profiling Reveals Aggravated Non-Alcoholic Steatohepatitis in High-Fat High-Cholesterol Diet-Fed Apolipoprotein E-Deficient Mice Lacking Ron Receptor Signaling
Source: Metabolites. 2020 Aug 11;10(8):326. doi: 10.3390/metabo10080326 (PMC7464030; doi:10.3390/metabo10080326)
Supplement: Supplementary file 1 [file metabolites-10-00326-s001.pdf]

Table S1. List of liver, fecal, serum bile acid, and internal standards (IS) names and abbreviations.

Abbreviation	Bile Acid Name
12 KLCA	3α-Hydroxy-12 Ketolithocholic Acid
3, 7, 12 DHCA	3,7,12 Dehydrocholic acid
3,12-DKCA	3,12-Diketocholanic Acid
3,6-DKCA	3,6-Diketocholanic Acid
3,7-DKCA	3,7-Diketocholanic Acid
3-EpiDCA	5β-Cholanic Acid-3β, 12α-diol
5β-CA-3a,6a-diol-7-one	5β-Cholanic Acid-3α, 6α-diol-7-one
5β-CA-7a-ol-3-one	5β-Cholenic Acid-7α-ol-3-one
8(14), 5β-CA-3α, 12α-diol	8(14), 5β-Cholenic Acid-3α, 12α-diol
7 KLCA	3α-Hydroxy-7 Ketolithocholic Acid
7, 12 DKCA	3α-Hydroxy-7,12-Diketocholanic Acid
9(11)-CA	9(11), (5β)-Cholenic Acid-3α-ol-12-one
ALCA	Allolithocholic Acid
AlloCA	Allocholanic acid
ApoCA	Apocholic acid
CA	Cholic acid
CDCA	Chenodeoxycholic acid
DCA	Deoxycholic acid
DHCA	3-Dehydrocholic Acid
GDCA	Glycodeoxycholic acid
GLCA	Glycolithocholic Acid
HCA	Hyocholic acid
HDCA	Hyodeoxycholic acid
IsoDCA	Isodeoxycholic Acid
IsoLCA	Isolithocholic Acid
KCA	3-Ketocholanic Acid
LC	Lithocholic acid
LCA	Lithocholenic acid
MuriDCA	Murideoxycholic acid
MuroCA	Murocholic Acid
norDCA	3α,12α, 23-Nordeoxycholic Acid
TCA	Taurocholic acid
TCDCA	Taurochenodeoxycholic acid
TDCA	Taurodeoxycholic acid
THCA	Taurohyocholic Acid
THDCA	Taurohyodeoxycholic Acid

TLCA	Taurolithocholic Acid
TUCA	Tauro-ursocholanic Acid
TUDCA	Tauroursodeoxycholic acid
TαMCA	Tauro-β-muricholic acid
TβMCA	Tauro-α-muricholic acid
TωMCA	Tauro-ω-muricholic acid
UCA	Ursocholanic Acid
UDCA	Ursodeoxycholic acid
αMCA	Alpha-Muricholic acid
βMCA	Beta- Muricholic acid
ωMCA	Omega- Muricholic acid
LC-d4 (IS)	Lithocholic Acid-d4
DCA-d4 (IS)	Deoxycholic Acid-d4
CDCA-d4 (IS)	Chenodeoxycholic Acid-d4
UDCA-d4 (IS)	Ursodeoxycholic Acid-d4
HDCA-d4 (IS)	Hyodeoxycholic Acid-d5
CA-d4 (IS)	Cholic Acid-d4
GLCA-d4 (IS)	Glycolithocholic Acid-d4
GCDCA-d4 (IS)	Glycochenodeoxycholic Acid-d4
GDCA-d4 (IS)	Glycodeoxycholic Acid-d4
GUDCA-d4 (IS)	Glycoursodeoxycholic Acid-d4
GCA-d4 (IS)	Glycocholic Acid-d4
TLCA-d4 (IS)	Taurolithocholic Acid-d4
TUDCA-d4 (IS)	Tauroursodeoxycholic Acid-d5
TCA-d4 (IS)	Taurocholic Acid-d4

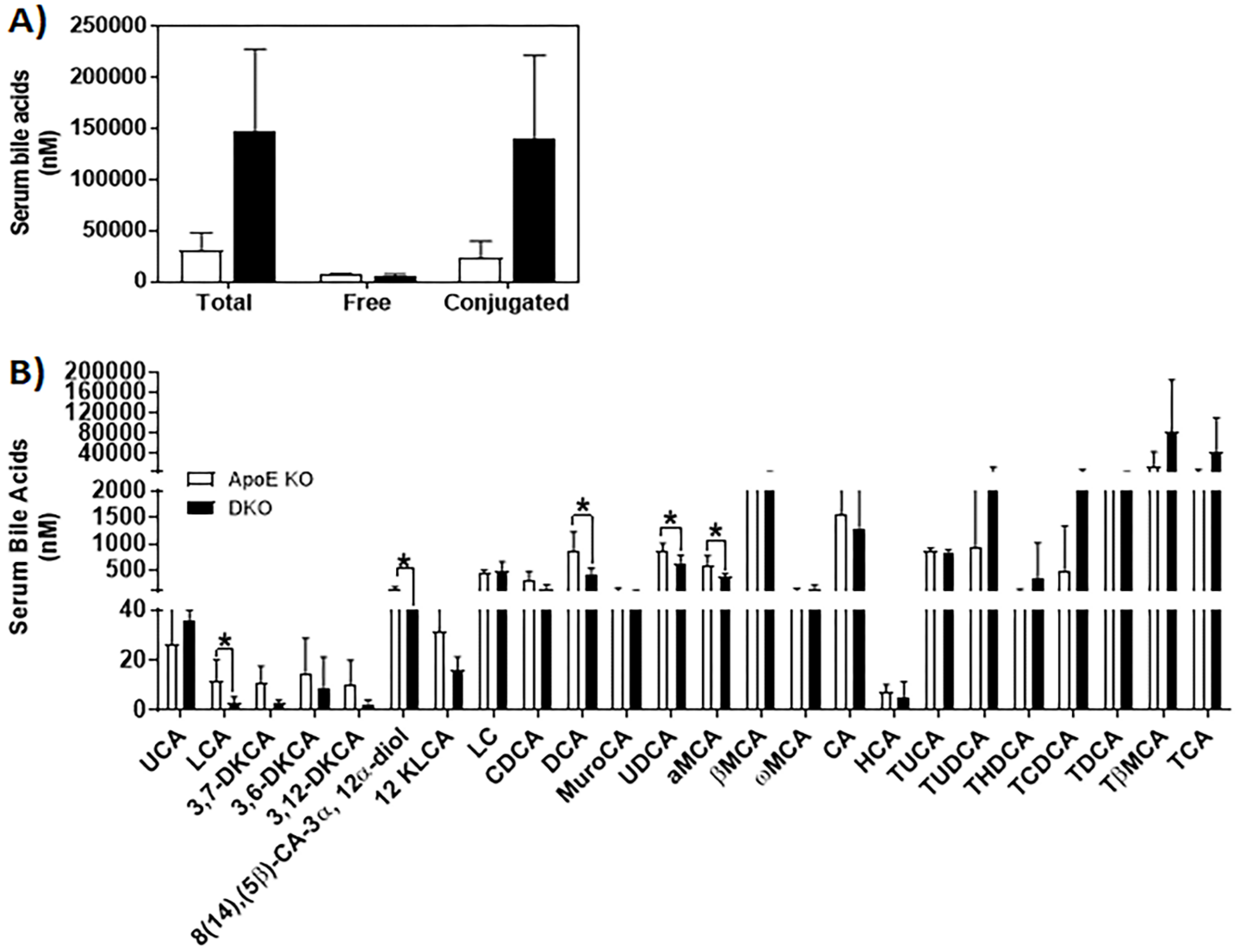

Figure S1. GC-MS analysis of bile acid concentration in serum derived from HFHCD-fed ApoE KO and DKO mice. **A.** Measured total, unconjugated, and conjugated bile acid pool in blood serum from HFHCD-fed animals (n = 6 per group). **B.** Concentration of individual bile acids in serum from HFHCD-fed ApoE KO and DKO mice (n = 6 per group). The data is presented as mean ± SEM, *$p < 0.05$, ** $p < 0.01$, *** $p < 0.001$.
